# Unconscious physiological response of healthy volunteers to dynamic respiration-synchronized couch motion

**DOI:** 10.1186/s13014-017-0925-6

**Published:** 2017-11-28

**Authors:** Alexander Jöhl, Marta Bogowicz, Stefanie Ehrbar, Matthias Guckenberger, Stephan Klöck, Mirko Meboldt, Oliver Riesterer, Melanie Zeilinger, Marianne Schmid Daners, Stephanie Tanadini-Lang

**Affiliations:** 1 Product Development Group Zurich, Department of Mechanical and Process Engineering, ETH Zurich, Zürich, Switzerland; 2 Department of Radiation Oncology, University Hospital Zurich, Zürich, Switzerland; 3 Institute for Dynamic Systems and Control, Department of Mechanical and Process Engineering, ETH Zurich, Zürich, Switzerland; 4 University of Zurich, Zürich, Switzerland

**Keywords:** Tumor tracking, Robotic couch, Motion sickness, Respiration pattern

## Abstract

**Background:**

Intrafractional motion can be a substantial uncertainty in precision radiotherapy. Conventionally, the target volume is expanded to account for the motion. Couch-tracking is an alternative, where the patient is moved to compensate for the tumor motion. However, the couch motion may influence the patient’s stress and respiration behavior decreasing the couch-tracking effectiveness.

**Methods:**

In total, 100 volunteers were positioned supine on a robotic couch, which moved dynamically and respiration synchronized. During the measurement, the skin conductivity, the heartrate, and the gaze location were measured indicating the volunteer’s stress. Volunteers rated the subjective motion sickness using a questionnaire. The measurement alternated between static and tracking segments (three cycles), each 1 min long.

**Results:**

The respiration amplitude showed no significant difference between tracking and static segments, but decreased significantly from the first to the last tracking segment (*p* < 0.0001). The respiration frequency differed significantly between tracking and static segments (*p* < 0.0001), but not between the first and the last tracking segment. The physiological parameters and the questionnaire showed mild signals of stress and motion sickness.

**Conclusion:**

Generally, people tolerated the couch motions. The interaction between couch motion and the patient’s breathing pattern should be considered for a clinical implementation.

**Trial registration:**

The study was registered at ClinicalTrials.gov (NCT02820532) and the Swiss national clinical trials portal (SNCTP000001878) on June 20, 2016.

**Electronic supplementary material:**

The online version of this article (10.1186/s13014-017-0925-6) contains supplementary material, which is available to authorized users.

## Background

In radiotherapy, the intrafractional motion of tumors can become a substantial uncertainty. For example, the motion of lung tumors may reach a peak-to-peak amplitude of up to 24 mm [1] or even 38 mm [2]. Liver tumors may reach 34 mm [3]. There are several approaches to mitigate this uncertainty [4], the conventional approach being the target volume expansion to cover all the possible positions of the tumor. While this approach ensures the dose coverage of the tumor, it increases the dose to surrounding healthy tissue. Alternative approaches are gating and dynamic tumor tracking techniques. In gated treatment, the tumor motion or its surrogate is monitored and the radiation beam is only switched on e.g. during a specific respiration phase. Dynamic tumor tracking techniques compensate the tumor motion continuously by moving the beam (CyberKnife [5], Vero [6]), modifying the beam (MLC tracking [7]) or counter-steering the patient with the robotic couch, which is called couch-tracking.

The advantages of couch-tracking are 1) it can be performed on a conventional linear accelerator as opposed to beam tracking and 2) it does not disturb the beam as opposed to MLC tracking. However, couch-tracking may influence the respiration of the patient or may induce stress or motion sickness, since the patient is being dynamically shifted with the couch during the treatment. Generally, the patient’s respiration influences the motion of tumors in the thorax or upper abdomen. During couch-tracking, the tumor motion is compensated by the motion of the robotic couch. However, the couch motion may also interact with the patient and influence the patient’s respiration, thus possibly reducing the couch-tracking effectiveness.

So far, three studies have been performed evaluating the behavior of volunteers or patients on a moving couch. Sweeney et al. [8] conducted a study with ten healthy volunteers and 23 patients. They were positioned supine on a robotic treatment couch executing a predefined cyclical trajectory for 30 min. This procedure was repeated once on a different day. The study endpoint was the procedure termination when the patients expressed the need to stop or to administer anti-nausea agents. D’Souza et al. [9] recruited 50 patients. They experienced several couch movement intervals that switched between static and dynamic conditions, in which the couch followed a pre-programmed trajectory. During each static segment, the ´Motion Sickness Assessment Questionnaire´ (MSAQ) [10] was administered. Wilbert et al. [11] performed a study with 15 healthy volunteers on a robotic couch, which counter-steered the respiration of the volunteer. The focus was on changes in volunteers’ respiration patterns.

The current study with 100 volunteers considered both the influence of respiration-driven couch motion on the respiratory pattern and the volunteers’ tolerance. The measurement alternated between static and tracking conditions in order to show whether volunteers got accustomed to tracking. In addition to the MSAQ, physiological signals (heartrate, skin conductivity, and eye motion) were recorded for an objective assessment of the mental state of the volunteers.

## Methods

The prospective study was designed such that each volunteer was placed on the couch only once and he/she experienced one sequence of couch motion conditions. The sequence consisted of three segments under tracking conditions (the couch moved according to volunteer’s respiration) and between these segments the couch did not move. The last segment consisted of a couch movement along a predefined trajectory independent of the volunteer’s respiration.

### Study population

Healthy, German-speaking volunteers between 18 and 100 years were eligible. Exclusion criteria were: known or suspected non-compliance, drug or alcohol abuse, the inability to follow the procedures, and a body weight above 200 kg. Informed consent was obtained from each volunteer prior to study entry.

### Study protocol

The study protocol was approved by the local ethics committee and was defined in accordance with the precepts in the ´Declaration of Helsinki´. The study was registered at ClinicalTrials.gov (NCT02820532) and the Swiss national clinical trials portal (SNCTP000001878).

### Couch tracking system

**Fig. 1:**
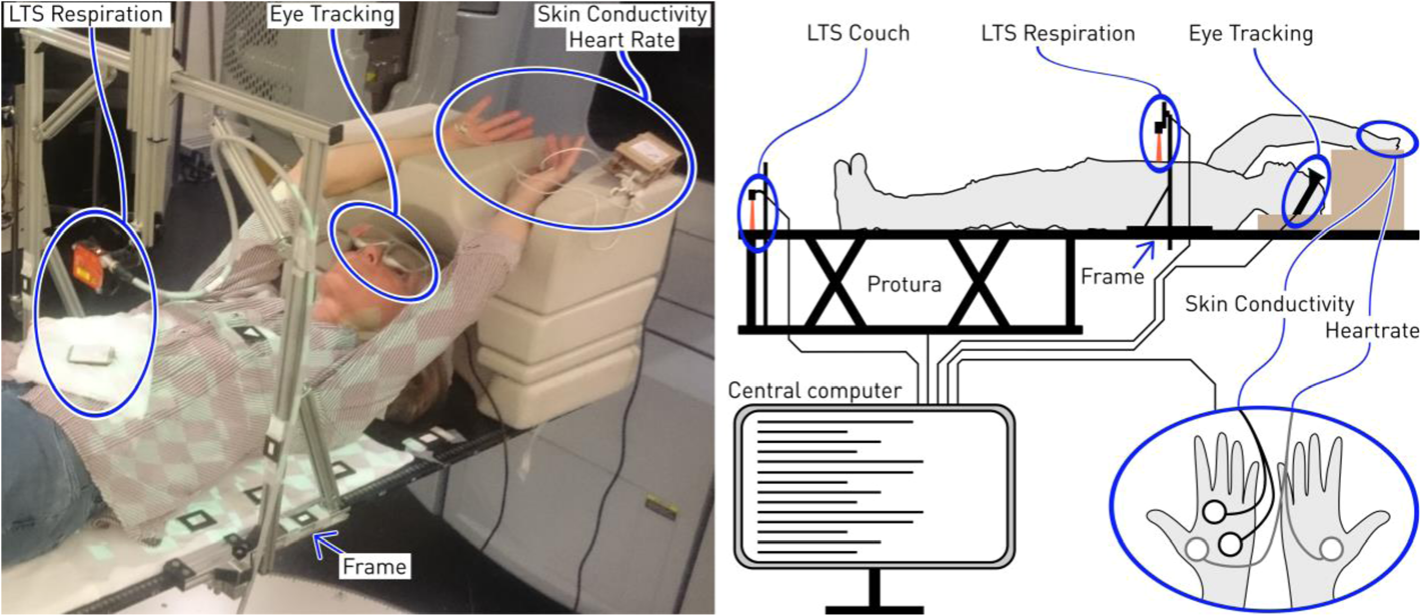
The volunteers were positioned supine with arms up. The left panel shows a volunteer with the sensors attached (blue ellipses). The right panel shows the schematic connection and placement of the sensors. The ellipse with two hands shows the placement of the electrodes for heartrate and skin conductivity measurements. LTS: laser triangulation system

The tracking system [12] consists of 1) the robotic couch, the Protura (CIVCO Medical Solutions, USA), 2) the sensors measuring the respiration and the couch position, and 3) the computer implementing the couch-tracking control (Fig. 1). The Protura has a position range of ±50 mm in SI, ±25 mm in LR and ±25 mm in AP direction. The Protura maximum speed and acceleration are 15 mm/s and 45 mm/s^2^, respectively, when simultaneously moving in the SI and AP directions [13]. For the same motion type, the Protura can compensate sinusoidal motion well up to 0.2 Hz. The respiration was measured using a laser triangulation system (LTS) (optoNCDT 1302, Micro Epsilon Messtechnik GmbH & Co. KG, Germany). The LTS was mounted on a frame that was fixed to the couch and measured the external chest or belly position. The frame allowed adapting the LTS position for the different volunteers. The LTS had a latency of about 2 ms and the low-pass filter of the LTS signal introduced a further 32 ms. The Protura couch showed a latency of about 100 ms. We did not use a prediction filter, since the focus of the study was not on the motion compensation performance. Additionally, no action was taken if the couch was too slow to follow the respiratory motion. We aimed to keep the system as simple as possible for reliability. Also, there was no training of the motion compensation during the initial static segment. Note that the measurements were carried out on a clinically used radiotherapy system in the hospital.

### Motion trajectory

The respiration of the volunteers was measured externally on the chest or abdomen in anterior-posterior (AP) direction, see section Couch tracking system. A fictitious tumor was assumed to move dependent on the respiration. The tumor motion was assumed to be two-dimensional (superior-inferior (SI) and AP) and linearly correlated to the external motion signal. The external motion’s amplitude varied among the volunteers due to different body sizes or measurement locations. To ensure that the couch stayed inside its position range, the tumor motion amplitudes across the volunteers had to be limited. Therefore, the peak-to-peak motion amplitude was normalized (for details see Additional file 1) and then multiplied by 2.86 mm for AP and 10.67 mm for SI. The LR motion was omitted due to being minimal in many patients. These numbers were chosen to approximate the average motion amplitudes as given in [1–3], while respecting the limits of the motion range of the robotic couch. The normalization value was the mean amplitude of the external motion during the initial segment.

### Measurement sequence

All volunteers were positioned supine with arms up on the treatment couch (Fig. 1).

**Fig. 2:**
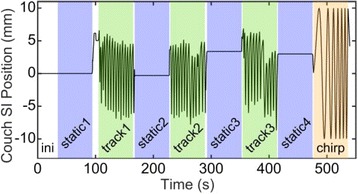
The measurement sequence of one volunteer. The line shows the superior-inferior (SI) position of the couch. The first interval (ini (white)) initializes the couch-tracking system. The colored intervals indicate the three segments: static (blue), track (green) and chirp (orange). During the track segments, the couch moved in accordance with the volunteer’s respiration. During the chirp segment, the couch moved sinusoidally independent of the respiration

The measurement sequence consisted of eight segments of three types: static, tracking and chirp (Fig. 2). After initialization, the couch was static for 1 min, then it switched to tracking (track) for 1 min and then back to static. This procedure was repeated twice and the sequence ended with a chirp segment. During the static segments, the couch kept its last position. During the tracking segments, the couch compensated the motion of the fictitious tumor. During the chirp segment, the couch followed a pre-programmed sinusoid whose frequency increased over time. The start of a tracking segment was synchronized with the end-expiration, when the speed of the fictitious tumor was minimal. During the complete sequence the gantry was in 0° position and did not move to avoid collision with the frame for the respiration measurement (Fig. 1).

### Physiological signals

#### Skin conductivity

The electrical conductivity between two electrodes on the left palm of the volunteer was measured (Fig. 1). The electrical conductivity depends on the humidity (sweat production) of the skin, which is influenced by the sympathetic nervous activity. Therefore, arousal or relaxation of the volunteer can be detected via the skin conductivity [14]. The hardware was the BITalino [15] (PLUX wireless biosignals S.A., Portugal). The analog signals of the sensors were converted to digital signals (10 bits) and sent to the central computer.

#### Heartrate

On each palm of the volunteer one electrode was attached (Fig. 1), which collected electrocardiogram data. The hardware used was also the BITalino.

#### Eye tracking

The volunteer wore eye-tracking glasses (SensoMotoric Instruments GmbH, Germany), which measured the gaze location and tracked the opening / closing of the eyes (Fig. 1).

#### Questionnaires

Each volunteer filled out two questionnaires (for details see Additional file 1): one before and one after the measurement. The first questionnaire asked for gender, age, weight, and history of motion sickness. The second questionnaire was the MSAQ described by Gianaros et al. [10], which allows the subjective assessment of motion sickness by volunteers. The statement scores of the MSAQ were grouped and averaged resulting in four scores, termed gastrointestinal, central, peripheral, and sopite related. The lowest possible score was one and the highest possible score nine, indicating the highest agreement with the respective statement.

#### Analyses

The respiration amplitude and frequency, the skin conductivity, the heartrate, and the distance from the overall average gaze location were analyzed. For each volunteer, these characteristic parameters were normalized by the volunteer’s overall mean value of the respective signal. The average over each segment was calculated resulting in eight values, assessed during four static segments, three tracking segments and one chirp segment. Note that the normalized skin conductivity values were offset, such that for all volunteers the first static segment value was zero. This reduces the base skin conductivity variation among the volunteers.

Two common hypotheses for all characteristic parameters were, 1) that the average of the values for the tracking segments was different from the static segments and 2) that the last tracking segment differed from the first tracking segment. The two-sided Wilcoxon sign rank test (significance level *p* < 0.05) was used to verify these hypotheses.

## Results

### Study population

One hundred healthy volunteers were included in the study (67 male, 33 female), (one male was excluded due to non-compliance, because he intentionally increased his respiration amplitude during tracking segments). Additionally, some measurements had to be excluded due to technical problems: The heartrate data had to be excluded for 21 volunteers due to poor signal-to-noise ratios. The skin conductivity data of 20 volunteers had to be excluded due to the signal hitting the sensor saturation limits (16 lower limit, 4 upper limit), and the eye-tracking data of four volunteers had to be excluded due to improper placement of the eye-tracking glasses. The volunteers’ age distribution ranged from 23 to 84 with a median of 32 years (Additional file 1: Figure S3). The resultant peak-to-peak amplitudes of the fictitious tumor ranged from 8 mm to 22 mm with a median of 14 mm. The respiration frequencies among the volunteers ranged from 0.07 Hz to 0.48 Hz.

### Motion sickness history of the volunteers

About half (56 of 100) of the volunteers reported to have experienced motion sickness in their life at least once. While 12 of 100 volunteers experienced motion sickness only once or twice, 27 volunteers experience motion sickness at sea at least occasionally and 30 volunteers at least occasionally riding in a car on a winding road. One volunteer reported to always experience motion sickness in cars or at sea.

### Physiological measurements

The results of the hypotheses tests are summarized in Table 1. For each test, the *p*-value and the 95% confidence interval (ci_95_) are shown. Three tests showed highly significant differences. The respiration amplitude and skin conductivity significantly decreased between the first and the last tracking segment. On the other hand, the respiration frequency was significantly higher during tracking than during static segments. The heartrate did not show any significant differences. The eye-tracking showed significant differences, lower values during tracking but they increased from the first to last tracking segment.

**Table 1:** Results of the two-sided Wilcoxon sign rank tests

		average track vs. average static		track3 vs. track1	
	Number of included volunteers	*p*-value	ci_95_	*p*-value	ci_95_
Respiration amplitude	99	0.1	–	< 0.0001	[−0.04, −0.12]
Respiration frequency	99	< 0.0001	[0.08, 0.14]	1	–
Heartrate	78	0.11	–	1	–
Skin conductivity	79	0.46	–	< 0.0001	[−0.29, −0.41]
Eye-tracking	95	0.02	[−0.01, −0.1]	0.003	[0.04, 0.19]

Each row shows one respiration characteristic or the result of one physiological measurement. Each row shows two tests, the first compared the average values of the tracking and the static segments, while the second compared the first with the last tracking segment. For each test, the *p*-value and the 95% confidence interval (ci_95_) are shown

### Respiration characteristics

**Fig. 3:**
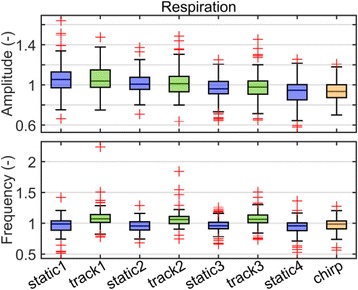
Box plots of the respiration amplitudes and frequencies. The values of each volunteer were normalized by his overall mean and then averaged over each segment resulting in eight values per volunteer. Each panel shows the distribution of the average amplitude and frequency of the segment over all volunteers

The median respiration amplitude (Fig. 3, upper panel) continuously decreased over the entire measurement time. On average over all segments, 57 volunteers showed an increase of the respiration amplitude during the tracking segments compared to the static segments. The median of the respiration frequency (Fig. 3, lower panel) always increased when the couch was tracking. However, during the chirp the median respiration frequency did not increase as much as during tracking.

### Skin conductivity

**Fig. 4:**
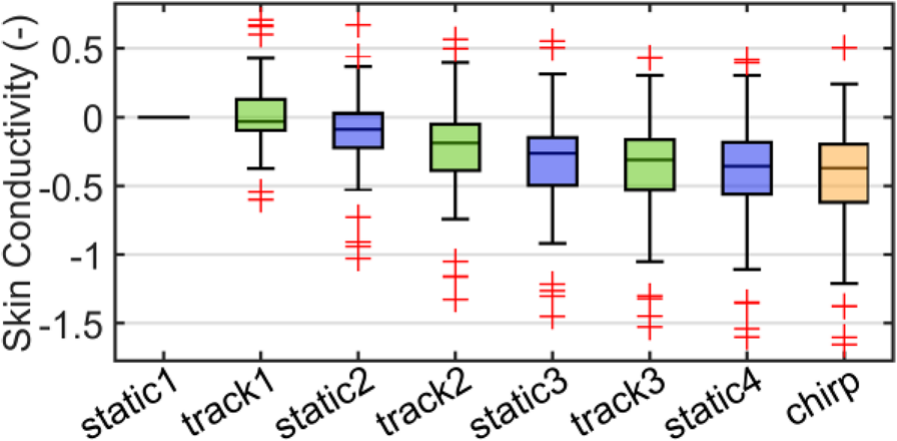
The box plot of the skin conductivity. The skin conductivity values of each volunteer were normalized by his overall mean and then averaged over each segment resulting in eight values per volunteer. Each panel shows the distribution of the segment averaged skin conductivity over all volunteers

The median skin conductivity decreased continuously (Fig. 4). The difference between the first tracking segment and the average of the first two static segments was significant (*p* < 0.0001, *ci*_95_ = [0.02, 0.07]). The chirp segment did not influence the median skin conductivity.

### Eye tracking

The eye tracking data showed significantly lower variation (segment mean distance of gaze location to overall average gaze location) of the gaze location during the tracking segments than during the static segments. However, the gaze location varied significantly more during the last tracking than during the first tracking segment.

### Motion sickness assessment questionnaire

**Fig. 5:**
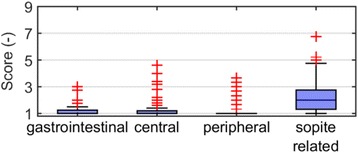
Average scores of the final questionnaire statements grouped into four motion sickness components. The higher the score, the higher the agreement with the statements corresponding to the given motion sickness component

The scores were low except for ‘sopite related’ statements, where the scores were higher but also more spread than for the other cases (Fig. 5). However, all four scores showed a few outliers. We have not found any correlations between these scores and motion sickness history or physiological measurement data (for details see Additional file 1).

## Discussion

Couch-tracking has the potential to reduce the treatment margins, which may lead to reduced side effects. However, one concern is the interaction between the patient and the moving couch. Here, we investigated whether the couch motion influences the respiration pattern of the volunteers and whether the couch motion induces any stress or motion sickness in the volunteers.

On average, the respiration frequency was significantly higher during tracking than during static segments. This increase is a potentially negative effect for couch-tracking, since it might cause increased compensation errors. However, during the final segment, when the couch moved along a predefined trajectory, the increase was not observed. Therefore, the increase is either due to a property of the feedback of the tracking system or of the respiration measurement. For many volunteers, the respiration measurement exhibited small oscillations above 1 Hz (for details see Additional file 1: Figure S7), which became substantial during expiration. In [11], the significant frequency increase may not have been found, due to the small number of volunteers (15) or because their respiration measurements did not show these oscillations. The couch compensated these oscillations causing a trembling couch motion, which the volunteer might perceive as uncomfortable. Therefore, the volunteers may have tended to spend less time in the expiration. This possibility has to be investigated further, but one solution might be to use low-pass filters to mitigate these oscillations at the cost of increasing the lag time of the couch-tracking system. The flexibility of the couch and the volunteers’ bodies might lead to the occurrence of a resonant frequency, which is higher than the respiration frequency. The noise of the LTS measurement may contain frequencies in the range of the resonant frequency and together with the feedback control, it might have led to the excitation of this resonant frequency.

The respiration amplitude did not change when the couch switched between the tracking and the static segments. However, it continuously decreased over the entire experiment. This decrease may be explained by the relaxation of the volunteers. This decrease may also cause a decrease in the tumor motion amplitude. Consequently, this relaxation could be exploited by having patients rest on the couch a few minutes before starting the treatment, which results in a smaller tumor motion amplitude and in turn may lead to smaller compensation errors. In [11], the mean amplitude over the first, the middle, and the final ten respiration cycles were computed during a five-minute measurement with the couch moving according to the respiration. For half of the volunteers, the authors found that the respiration amplitudes decreased from the first ten to the last ten cycles. This agrees with our observations. Our results additionally show that the decrease of the respiration amplitude was not affected by the couch’s switching between static and tracking conditions.

In the second part, we investigated whether the couch motion induces stress or motion sickness in the volunteers. The overall experience of the couch-tracking was evaluated using the MSAQ. The first three scores show very little evidence of motion sickness, but the fourth score (sopite related) showed higher values than the other scores. This fourth score included statements on tiredness and sleepiness, therefore, higher values of the fourth score could point to relaxation instead of motion sickness (for details see Additional file 1). The majority of the examinations took place in the evening. However, as there were outliers in all scores, a small fraction of patients might need closer observation in couch-tracking. The MSAQ was also applied in [9], but the authors considered the overall score. Their resulting scores were generally low, which coincides with our results. Similar results were reached in [8], where none of the subjects needed to interrupt a 30-min session of lying on a moving couch.

The skin conductivity results showed the overall relaxation (significant difference between first and last tracking segments), except for the first tracking segment, which showed a significant increase in skin conductivity emphasizing the elevated mental strain at the beginning of tracking. During the subsequent tracking segments, the skin conductivity decreased. This observation can be explained by the tracking being a new experience (first tracking segment), to which people get accustomed over the next segments. The question remains, whether people remain accustomed to couch-tracking between treatment fractions of consecutive days.

The eye-tracking showed smaller deviations from the mean gaze location during tracking segments than during static segments. The volunteers tended to focus their gaze, when the couch was moving, possibly to look for stability (analogously to looking at the horizon while balancing). However, the increase of the gaze deviations from the first tracking segment to the last tracking segment indicates that the volunteers become accustomed to tracking (for details see Additional file 1). The heartrate did not show any significant variation due to couch-tracking or overall relaxation. Since both the skin conductivity and the heartrate did not show any consistent variations with the respiration characteristics, it does not seem possible to predict the respiration characteristics using these physiological signals.

The stress of the volunteers was only slightly increased due to the couch motion (more focused gaze, increased skin conductivity). The physiological measurements agreed with the results of the MSAQ, in so far that both showed small signals of stress.

The equipment to perform the physiological measurements might have influenced the results, because, such additional devices might increase the stress level of the volunteers. However patients might have a higher level of stress, which could influence the ability to tolerate couch motion. Additionally, the gantry was static during the study, but sudden changes in the motion of the gantry during treatment could influence the stress of the patient. Patients might also have respiration patterns that are rather different from those of healthy volunteers. Additionally, the fictitious tumor motion model consisted only of a straight line. Such a model does not cover all real tumor motion trajectories.

The median age of the volunteers was 32 years, which does not reach the typical age of cancer patients, but there were 14 volunteers above the age of 60 years. However, we did not find any relationships between the aforementioned results and the volunteers’ age.

The breathing amplitudes were normalized to ensure that the fictitious tumor position stayed inside allowed motion range for the Protura. However, the normalization values were computed only on the initial static segment. Therefore, the normalization values were only approximations, which led to some variation of the resultant fictitious tumor motion amplitude (Additional file 1: Fig. S4). The LR motion was omitted due to being generally small. The LR motion could alter the patient response, since the body could rotate around the SI axis, which could be a different sensation. However, since only few patients have considerable LR motion, we neglected the LR motion.

## Conclusion

The significant increase in respiration frequency clearly shows that the patient’s respiration and the couch movement interact. Therefore, further investigations are needed to quantify the impact of this change in respiration pattern on the couch-tracking performance. As the volunteers seem to quickly grow accustomed to the tracking motion (shown by a significant decrease of respiration amplitude and skin conductivity) and they only show very mild symptoms of stress or motion sickness, the viability of couch-tracking is indicated.

## Additional file

Additional file 1: The questionnaires and their results. (PDF 1275 kb)

## Abbreviations

AP: Anterior-posterior
ci_95_: 95% confidence interval
LR: Left-right
LTS: Laser triangulation system
MLC: Multileaf collimator
MSAQ: Motion sickness assessment questionnaire
SI: Superior-inferior
