# Supplementary material for: Unconscious physiological response of healthy volunteers to dynamic respiration-synchronized couch motion
Source: Radiat Oncol. 2017 Nov 28;12:189. doi: 10.1186/s13014-017-0925-6 (PMC5706399; doi:10.1186/s13014-017-0925-6)
Supplement: Additional file 1: — The questionnaires and their results. (PDF 1275 kb) [file 13014_2017_925_MOESM1_ESM.pdf]

Supplemental materials for “Unconscious physiological response of healthy volunteers to dynamic respiration-synchronized couch motion”

Authors

Alexander Jöhl[1,2], Marta Bogowicz[2,4], Stefanie Ehrbar[2,4], Matthias Guckenberger[2,4], Stephan Klöck[2,4], Mirko Meboldt[1], Oliver Riesterer[2,4], Melanie Zeilinger[3], Marianne Schmid Daners[1], Stephanie Tanadini-Lang[2,4]

[1] Product Development Group Zurich, Department of Mechanical and Process Engineering, ETH Zurich

[2] Department of Radiation Oncology, University Hospital Zurich

[3] Institute for Dynamic Systems and Control, Department of Mechanical and Process Engineering, ETH Zurich

[4] University of Zurich

Content

Authors 1
Content 1
1. Sheet for Questionnaire 1 2
2. Sheet for Questionnaire 2 3
3. Age histogram 4
4. Fictitious tumor motion amplitudes 4
5. Normalization of external respiratory signal 4
6. Histograms of respiration characteristics and physiological signals 5
7. Small oscillations of respiration measurement 6
8. Eye tracking results 6
9. Score histograms of Questionnaire 2 7
10. Questionnaire 2 scores and motion sickness history 8
11. Respiration characteristics and scores of questionnaire 2 10
12. Physiological measurements and scores of Questionnaire 2 11
References 13

1. Sheet for Questionnaire 1

Fragebogen 1 **1**

Versuchspersonnummer: ____________

Geschlecht: ☐ weiblich ☐ männlich

Körpergewicht (in kg): ______________

Alter (in Jahren): ______________

Fragen zu Ihrer Vorgeschichte

Wurde Ihnen schon einmal schlecht aufgrund von Bewegungen (auf dem Schiff, im Auto…)?

☐ Ja ☐ Nein

Wenn ja, wie oft?

☐ 1-2 mal
☐ manchmal bei bergigen Autofahrten
☐ manchmal bei Schifffahrten
☐ häufig bei bergigen Autofahrten
☐ häufig bei Schifffahrten
☐ immer bei bergigen Autofahrten
☐ immer bei Schifffahrten
☐ bei anderen Dingen, bitte Häufigkeit und bei was angeben:

Bitte nicht ausfüllen:

☐ Kopfabstand (cm): ____________

ETH zürich Fragebogen 1 pd|z

Fig. 1: The first questionnaire (in German), which was filled in by each volunteer before the measurement procedure.

The first part of the questionnaire shown in Fig. 1 asks for the gender of the volunteer (female, male), the body weight in kg, and the age in years. The second part asks for the history of motion sickness, whether it has happened before (yes, no), and if yes, how often. The possible answers to tick are:

- once or twice
- sometimes on a winding road
- sometimes on a boat
- often on a winding road
- often on a boat

- always on a winding road
- always on a boat
- Any other possible situations. In such a case, the volunteers were asked to describe the situation and write how often it happened.

2. Sheet for Questionnaire 2

Fragebogen 2 1

Versuchspersonnummer: ____________

Fragen zu Ihren Empfindung beim Versuch

Bitte bewerten Sie jede Aussage mit einer Nummer zwischen 1 und 9. Eine 1 bedeutet, dass Sie dieser Aussage gar nicht zustimmen und Ihr Zustand gar nicht beeinträchtigt ist. Eine 9 bedeutet, dass Sie dieser Aussage zustimmen und Ihr Zustand derart beeinträchtigt ist, dass das Experiment abgebrochen werden muss.

Mir wurde schlecht ____________

Mir wurde schwindlig ____________

Ich fühlte mich genervt / irritiert ____________

Ich habe geschwitzt ____________

Ich habe mich unwohl gefühlt ____________

Ich fühlte mich benommen ____________

Ich habe mich schläfrig gefühlt ____________

Ich hab kalten Schweiss gespürt ____________

Ich fühlte mich verwirrt ____________

Ich habe mich müde gefühlt ____________

Mir wurde übel ____________

Mir wurde heiss ____________

Es war Schwindel erregend ____________

Es hat sich angefühlt, als ob sich alles drehen würde ____________

Ich fühlte mich, als ob ich mich übergeben müsste ____________

Ich war beunruhigt ____________

ETH zürich Fragebogen 2 pd|z

Fig. 2: The second questionnaire (in German), which was filled in after the volunteers left the couch after the measurement procedure. This questionnaire is the German version of the questionnaire developed in [1].

The English version of the statements are (in the same order as shown in Fig. 2 and taken directly from [1]):

- I felt sick to my stomach (G)
- I felt faint-like (C)

- I felt annoyed/irritated (S)
- I felt sweaty (P)
- I felt queasy (G)
- I felt lightheaded (C)
- I felt drowsy (S)
- I felt clammy/cold sweat (P)
- I felt disoriented (C)
- I felt tired/fatigued (S)
- I felt nauseated (G)
- I felt hot/warm (P)
- I felt dizzy (C)
- I felt like I was spinning (C)
- I felt as if I may vomit (G)
- I felt uneasy (S)

The single characters in the brackets after each line indicate to which of the four dimensions of motion sickness the statement belongs:

- G: Gastrointestinal
- C: Central
- P: Peripheral
- S: Sopite-related

3. Age histogram

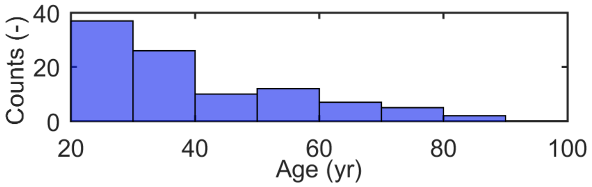

Fig. 3: Age histogram of all volunteers. The age range was 23 to 84 years and the median was 32 years.

4. Fictitious tumor motion amplitudes

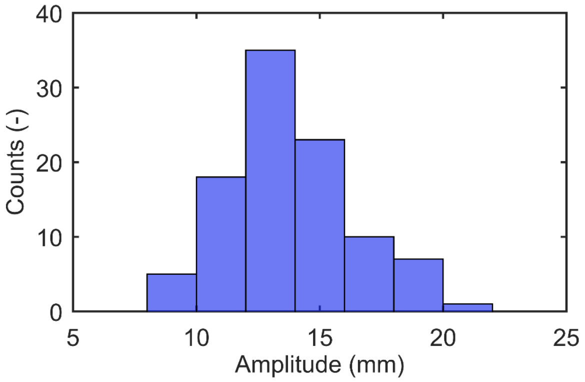

Fig. 4: Histogram of the overall mean peak-to-peak amplitudes of the fictitious tumors of the volunteers.

5. Normalization of external respiratory signal

The normalization of the peak-to-peak amplitude of the external respiration signal required an estimate of the actual peak-to-peak amplitude of the external signal as a normalization value. The

estimation approach was to detect the inspiration and expiration peaks and sequentially computing their differences. Then, the normalization value was the mean of these differences. However, the real-time detection of the peaks tended to lag behind the actual peaks, especially the inspiration peaks (Fig. 5). The detection approach was to do a linear fit of the last 0.8 s and consider the zero-crossings of the slope of the linear fit. This approach caused a systematic underestimate of the peak-to-peak amplitude. The underestimate explains the difference between the resulting median fictitious tumor motion peak-to-peak amplitude of 14 mm and the preset multiplication value of 11 mm for the fictitious tumor motion (see section Motion trajectory).

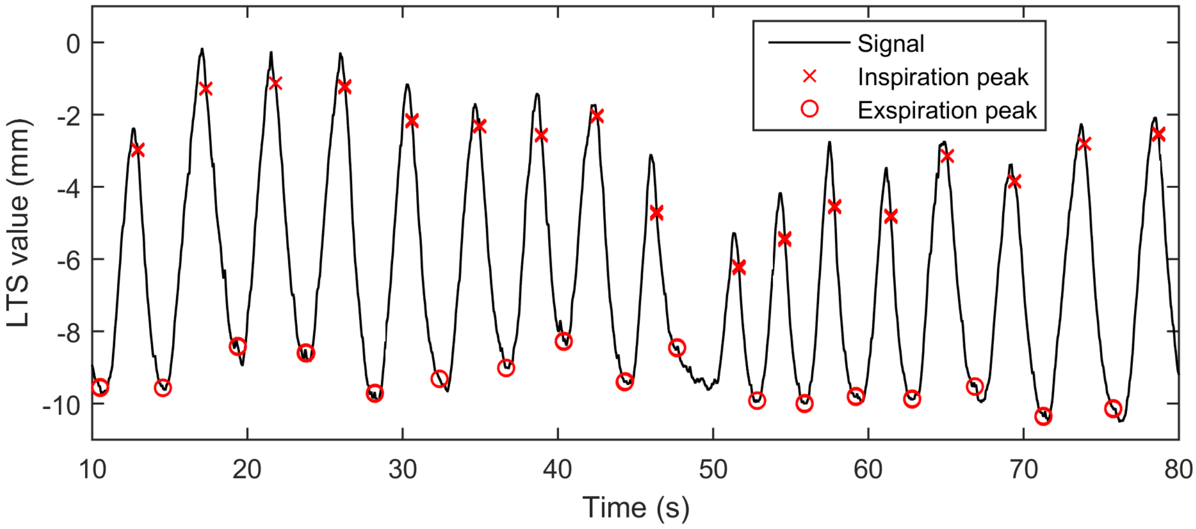

Fig. 5: Example for the peak detection results. The peak detection of the inspiration peaks lagged behind, which led to an estimate of the peak-to-peak amplitude smaller than the actual peak-to-peak amplitude.

6. Histograms of respiration characteristics and physiological signals

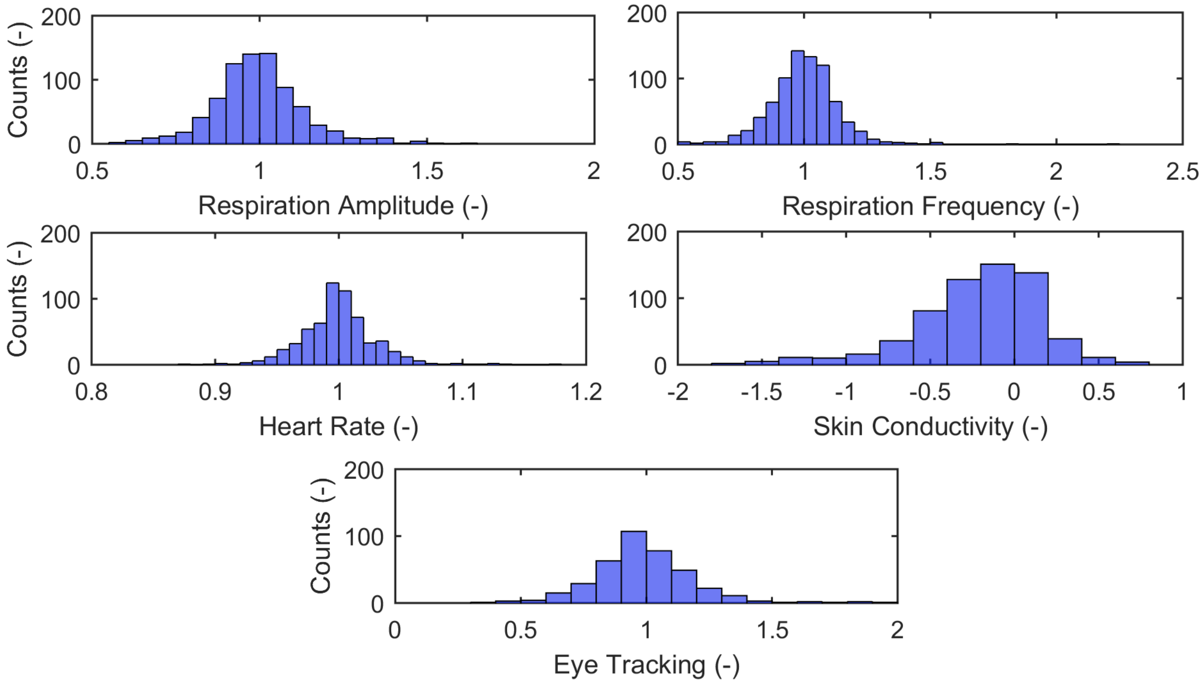

Fig. 6: The histograms of the respiration characteristics and the physiological signals after taking their respective mean values over all segments. There were eight values (three tracking, four static, and one chirp) per volunteer included. The histograms contain all segment mean values of each included volunteer, as they were used in the hypothesis tests. All values were normalized.

7. Small oscillations of respiration measurement

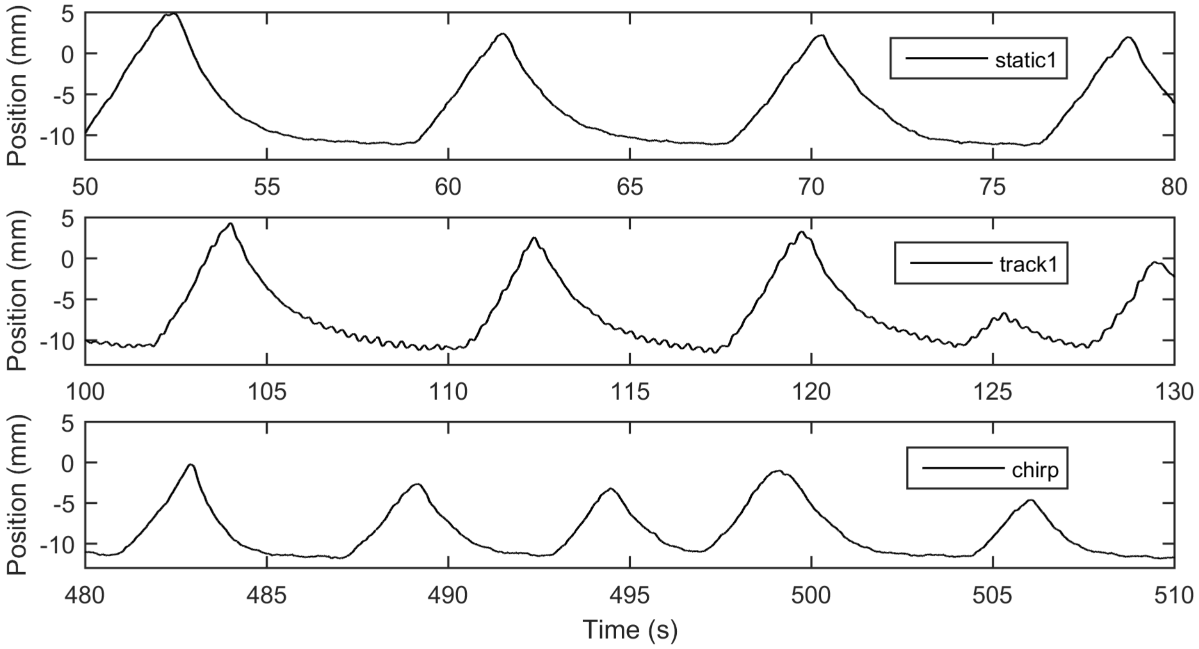

Fig. 7: The respiration measurements of one volunteer. Three short intervals taken from the first static segment, the first tracking segment, and the chirp segment. The middle panel shows the small oscillation (see Section 4. Discussion in the original manuscript) at frequencies above 1 Hz, which do not occur in the other segments. These small oscillations might be the cause of the respiration frequency increase during the tracking segment.

8. Eye tracking results

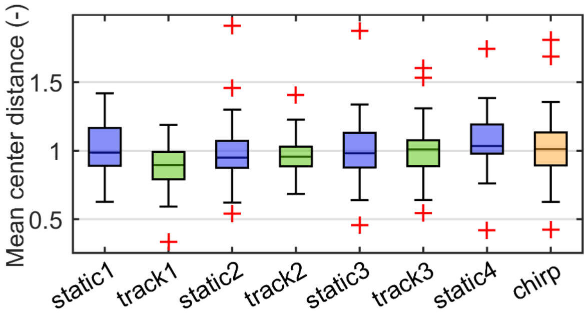

Fig. 8: Box plot of the mean center distances of the current gaze location. The center is defined as the average gaze location over the complete measurement procedure. For each segment and volunteer, the mean distance of the gaze location from the center was computed.

9. Score histograms of Questionnaire 2

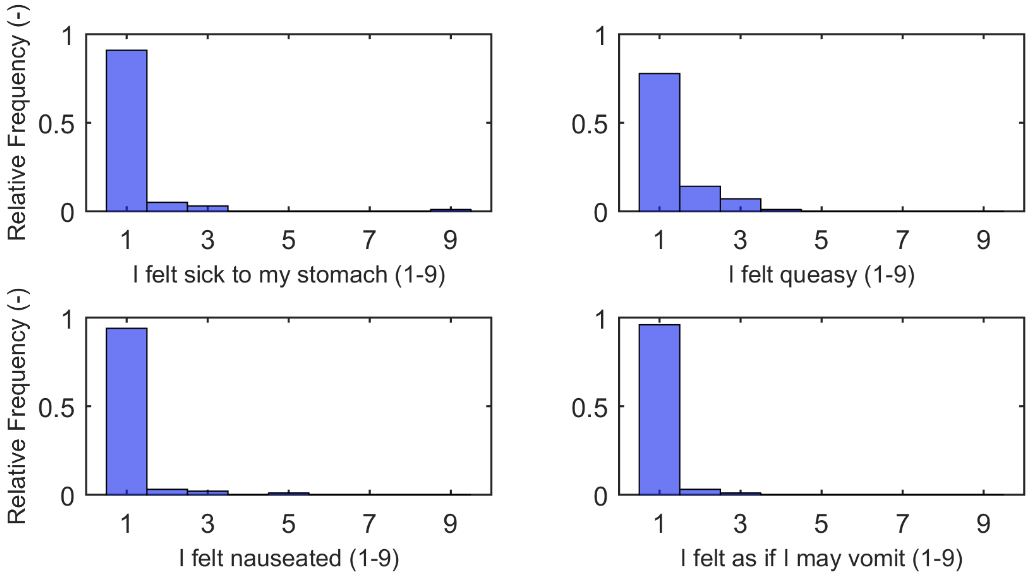

Fig. 9: Histograms of the scores for the statements of Questionnaire 2 associated with the gastrointestinal dimension of motion sickness [1].

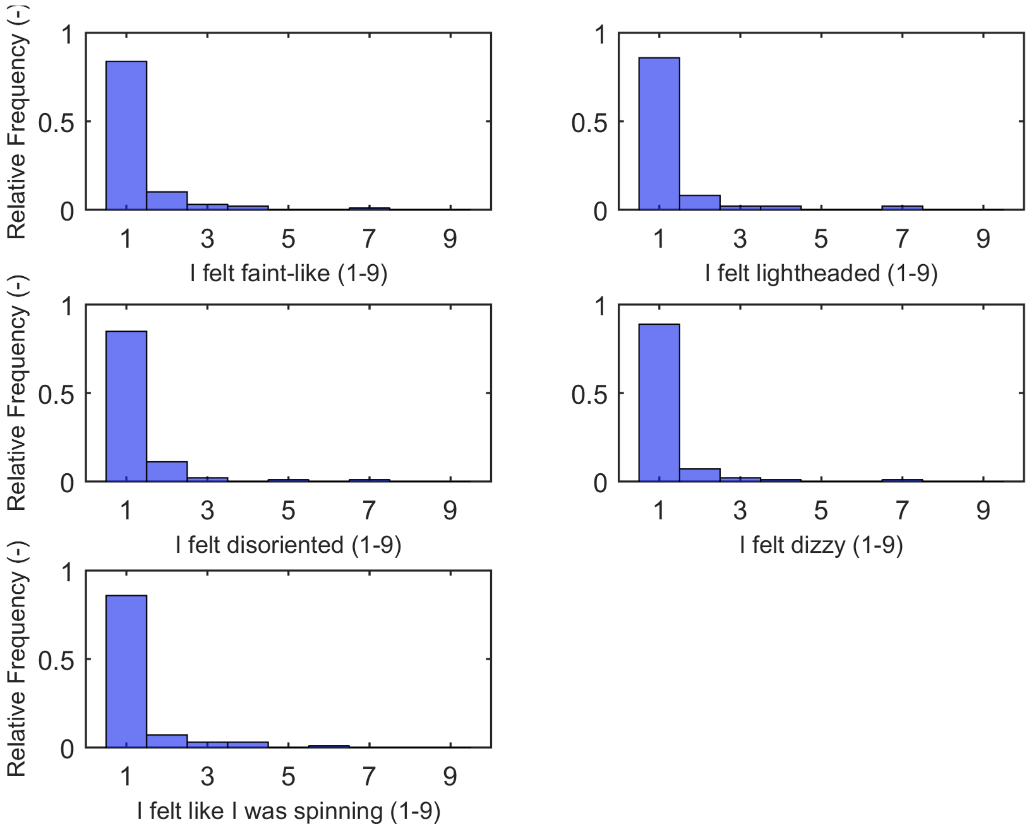

Fig. 10: Histograms of the scores for the statements of Questionnaire 2 associated with the central dimension of motion sickness [1].

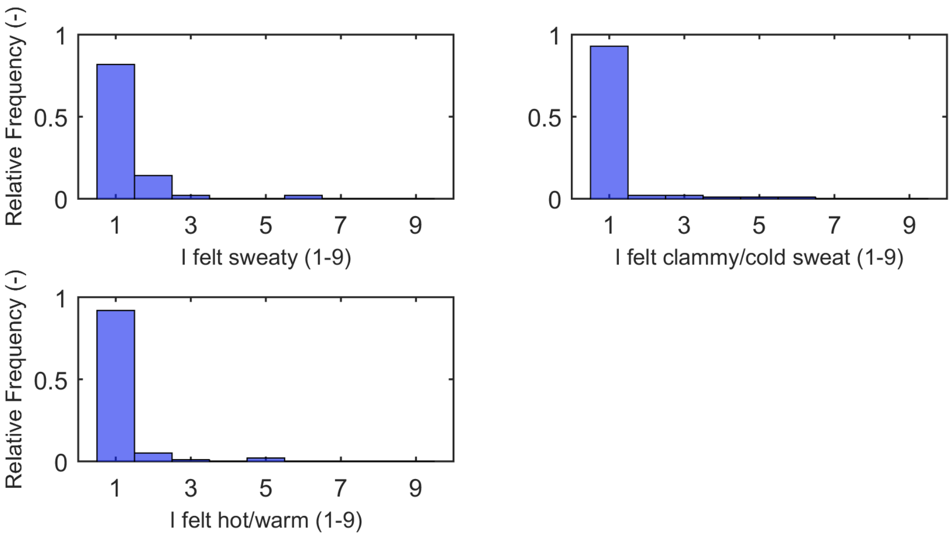

Fig. 11: Histograms of the scores for the statements of Questionnaire 2 associated with the peripheral dimension of motion sickness [1].

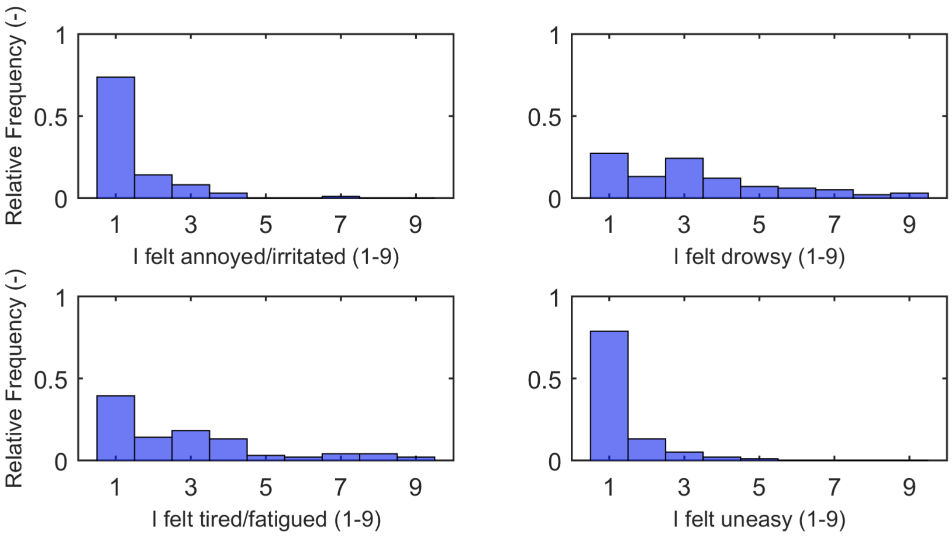

Fig. 12: Histograms of the scores for the statements of Questionnaire 2 associated with the sopite related dimension of motion sickness [1].

10. Questionnaire 2 scores and motion sickness history

The scores of Questionnaire 2 seem to be only very slightly dependent on motion sickness history. Volunteers with motion sickness history tended to report slightly higher scores.

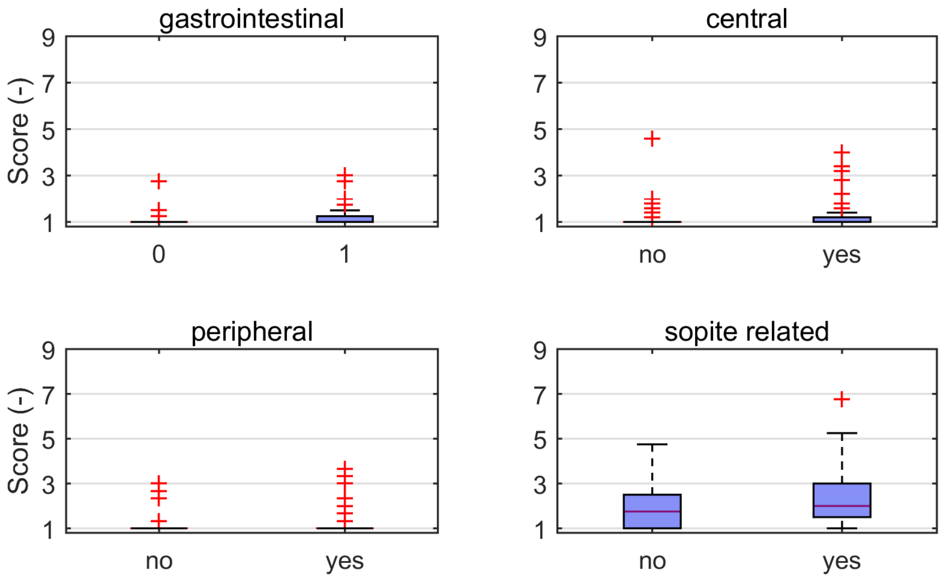

Fig. 13: Box plots of the four dimensions of motion sickness (Questionnaire 2). Each panel shows the scores (median, quartiles and whiskers, which include all data points below a given distance from the respective quartile) of volunteers that either reported in Questionnaire 1 to have experienced motion sickness before (yes) or did not (no).

11. Respiration characteristics and scores of questionnaire 2

The respiration characteristics (amplitude and frequency) do not seem to correlate to the scores of Questionnaire 2 (Fig. 14 and Fig. 15). The Pearson correlation coefficients are generally small and the corresponding p-values not significant.

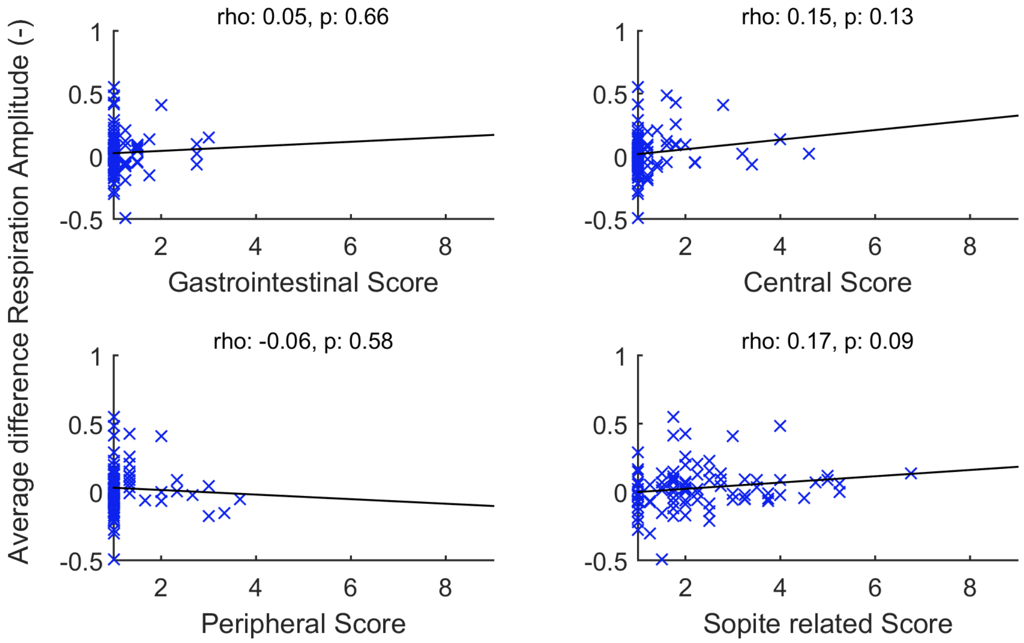

Fig. 14: Scatterplot of the average difference of the respiration amplitude between the static segments and the tracking segments and the corresponding score of Questionnaire 2. Each blue cross corresponds to one volunteer. Above each panel is the linear Pearson correlation coefficient (rho) with the corresponding p-value (p). The black line shows the best linear fit.

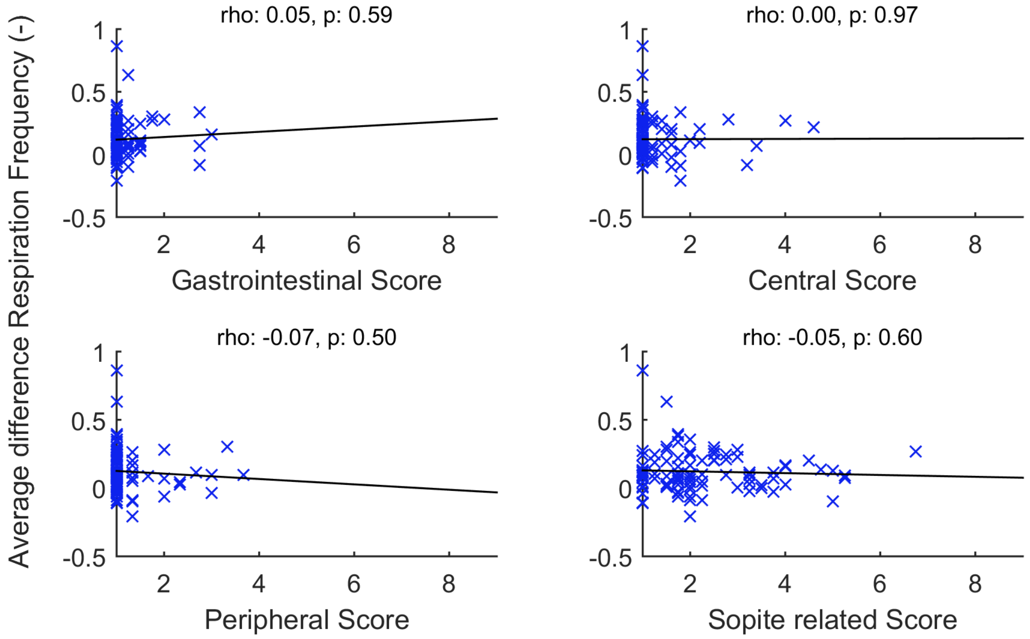

Fig. 15: Scatterplot of the average difference of the respiration frequency between the static segments and the tracking segments and the corresponding score of Questionnaire 2. Each blue cross corresponds to one volunteer. Above each panel is the linear Pearson correlation coefficient (rho) with the corresponding p-value (p). The black line shows the best linear fit.

12. Physiological measurements and scores of Questionnaire 2

The skin conductivity increase during the first tracking segment does not seem to correlate with Questionnaire 2 scores, see Fig. 16. The Pearson correlation coefficients are generally small and the corresponding p-values not significant.

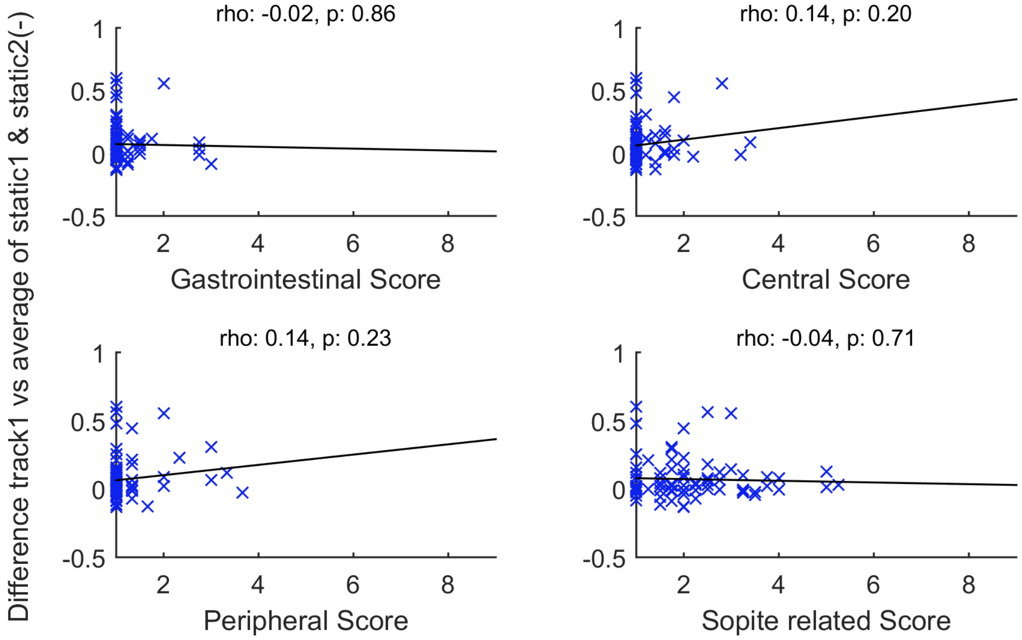

Fig. 16: Scatterplot of the difference of the skin conductivity during the first tracking segment and the mean of the first and second static segments, and the corresponding score of Questionnaire 2. Each blue cross corresponds to one volunteer. Above each panel is the linear Pearson correlation coefficient (rho) with the corresponding p-value (p). The black line shows the best linear fit.

The heartrate difference between tracking and static segments does not seem to correlate with Questionnaire 2 scores, see Fig. 17. The Pearson correlation coefficients are generally small and the corresponding p-values not significant.

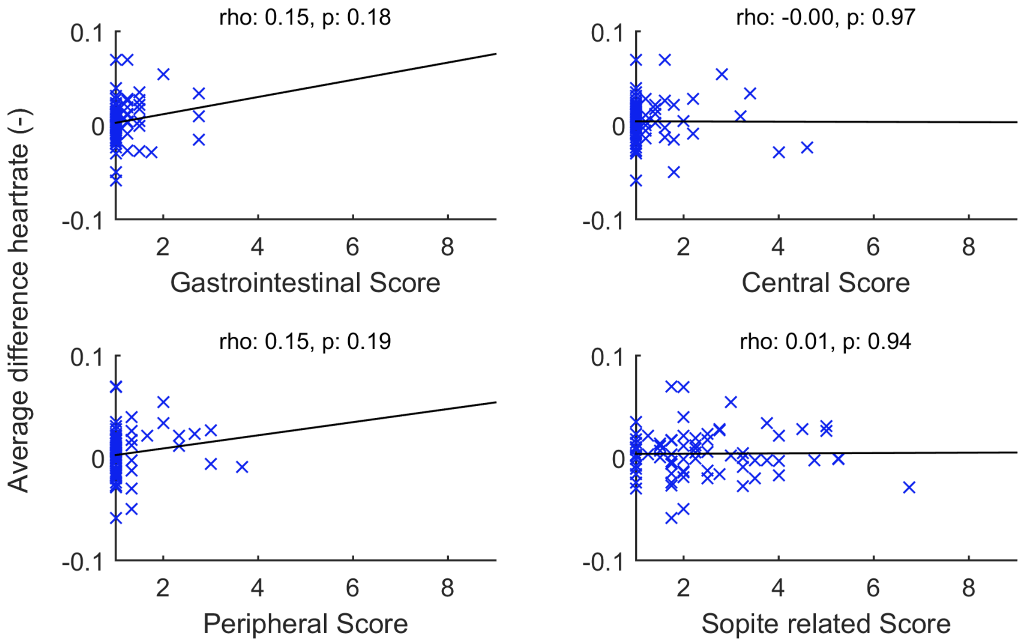

Fig. 17: Scatterplot of the average difference of the heartrate during the tracking segments and the static segments, and the corresponding score of Questionnaire 2. Each blue cross corresponds to one volunteer. Above each panel is the linear Pearson correlation coefficient (rho) with the corresponding p-value (p). The black line shows the best linear fit.

The gaze-point deviation difference between tracking and static segments does not seem to correlate with Questionnaire 2 scores, see Fig. 18. The Pearson correlation coefficients are generally small and the corresponding p-values not significant.

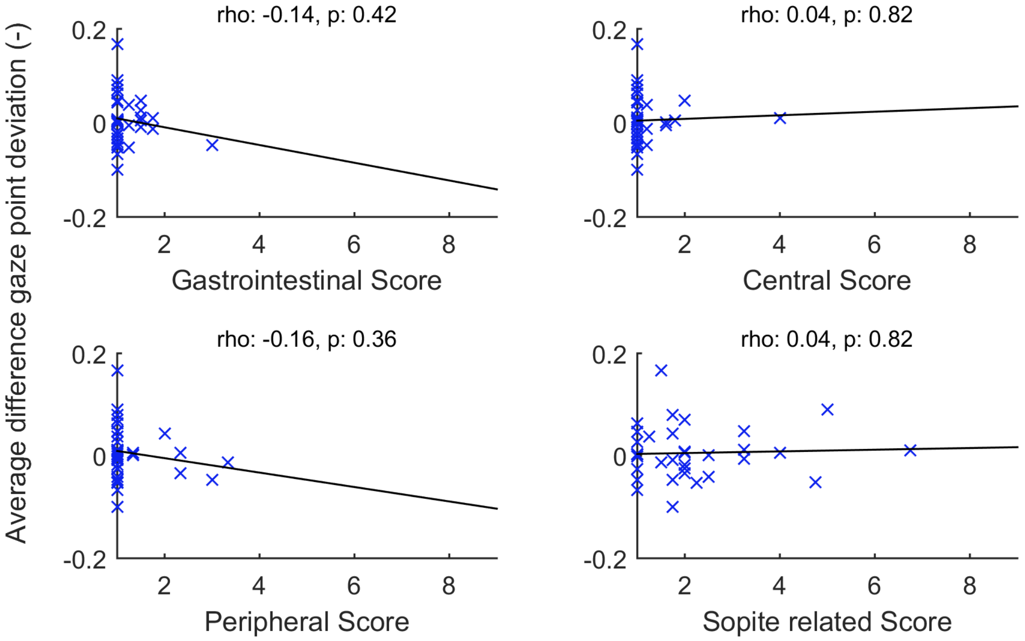

Fig. 18: Scatterplot of the average difference of the gaze-point deviation during the tracking segments and the static segments, and the corresponding score of Questionnaire 2. Each blue cross corresponds to one volunteer. Above each panel is the linear Pearson correlation coefficient (rho) with the corresponding p-value (p). The black line shows the best linear fit.

References

[1] Gianaros PJ, Muth ER, Mordkoff JT, Levine ME, Stern RM. A questionnaire for the assessment of the multiple dimensions of motion sickness. Aviation, space, and environmental medicine, 2001;72:115–119.
